# Sclerotic bone lesions as a potential imaging biomarker for the diagnosis of tuberous sclerosis complex

**DOI:** 10.1038/s41598-018-19399-7

**Published:** 2018-01-17

**Authors:** Susanne Brakemeier, Lars Vogt, Lisa C. Adams, Bianca Zukunft, Gerd Diederichs, Bernd Hamm, Klemens Budde, Kai-Uwe Eckardt, Marcus R. Makowski

**Affiliations:** 1 Department of Nephrology and Medical Intensive Care, Charité, Charitéplatz 1, 10117 Berlin, Germany; 2 Department of Radiology, Charité, Charitéplatz 1, 10117 Berlin, Germany; 3 King’s College London, Division of Imaging Sciences, Westminster Bridge Road, London, SE1 7EH United Kingdom

## Abstract

Tuberous-sclerosis-complex (TSC) is associated with a high lifetime risk of severe complications. Clinical manifestations are largely variable and diagnosis is often missed. Sclerotic-bone-lesions (SBL) could represent a potential imaging biomarker for the diagnosis of TSC. In this study, computed tomography (CT) data sets of 49 TSC patients (31 females) were included and compared to an age/sex matched control group. Imaging features of SBLs included frequency, size and location pattern. Sensitivities, specificities and cutoff values for the diagnosis of TSC were established for the skull, thorax, and abdomen/pelvis. In TSC patients, 3439 SBLs were detected, including 665 skull SBLs, 1426 thoracal SBLs and 1348 abdominal/pelvic SBLs. In the matched control-collective, 157 SBLs could be found. The frequency of SBLs enabled a reliable differentiation between TSC patients and the control collective with the following sensitivities and specificities. Skull: ≥5 SBLs, 0.783, 1; thorax: ≥4 SBLs, 0.967, 0.967; abdomen/pelvis: ≥5 SBLs: 0.938, 0.906. SBL size was significantly larger compared to controls (p < 0.05). Based on the frequency, size and location pattern of SBLs TSC can be suspected. SBLs may serve as a potential imaging biomarker in the workup of TSC patients.

## Introduction

Tuberous sclerosis complex (TSC) is a rare autosomal dominant disorder that affects approximately 1.5 million people worldwide with a birth incidence of 1 in 6000^1^. Between 70% to 80% of patients show no family history of TSC and have a sporadic mutation^2^. Genetic testing is positive in only up to 80% of patients with mutations in the TSC1 or the TSC2 genes which encode for the mTOR-regulated proteins hamartin or tuberin, respectively^2^. The clinical picture is determined primarily by the uncontrolled growth of mostly benign mesenchymal tumors in nearly all organ systems. Clinical manifestations of TSC are highly variable depending on age and organ manifestation of the respective patient^3^. As a consequence, the diagnosis of TSC can be difficult to establish or even be missed^4^. Approximately 50% of patients with TSC present with epileptic seizures in early childhood as a result of CNS involvement and diagnosis is normally confirmed without delay. In the other half of patients with TSC, diagnosis is mostly suspected only in case of organ complications such as acute AML bleeding^5^.

Current diagnostic guidelines are based on a typical combination of organ manifestations of the brain, kidney, skin, lung and heart (Supplementary Table)^5^. Even though TSC has been known for more than 130 years only a limited number of studies describing details of the phenotype are available, due to its low prevalence. Most publications are either case reports or include a relatively small study population^6–8^. However, a reliable diagnosis of TSC is of high importance for affected patients, especially if they are oligosymptomatic. Renal involvement with angiomyolipomas (AMLs) causes renal failure, which is the leading cause of death in adults with TSC^9,10^. Acute AML bleeding is also frequent in TSC patients with a lifetime risk of approximately 20%^11,12^.

Accumulating evidence indicates that organ manifestations of TSC (e.g. pulmonary, renal, cerebral and skin lesions) can be successfully treated with mTOR-inhibitors^11–16^. Given the combination of promising novel therapeutic strategies and a high lifetime risk of life-threatening complications, missed or delayed diagnosis should be avoided. A recent consensus conference emphasized this goal by defining the need for additional diagnostic biomarkers to improve the detection of patients with TSC^17^.

Previous studies have already suggested that sclerotic bone lesions (SBLs) are associated with TSC. However, the diagnostic potential of SBLs for the primary diagnosis of TSC has not been evaluated yet^2,7,18,19^.

The aim of the current study was to assess the potential of sclerotic bone lesions (SBLs) detected on CT as an additional imaging biomarker for the diagnosis of TSC. We compared the frequency, location and size of SBLs in the skull, thorax, abdomen/pelvis in TSC patients to matched healthy controls.

## Material and Methods

### Study population

**Figure 1:**
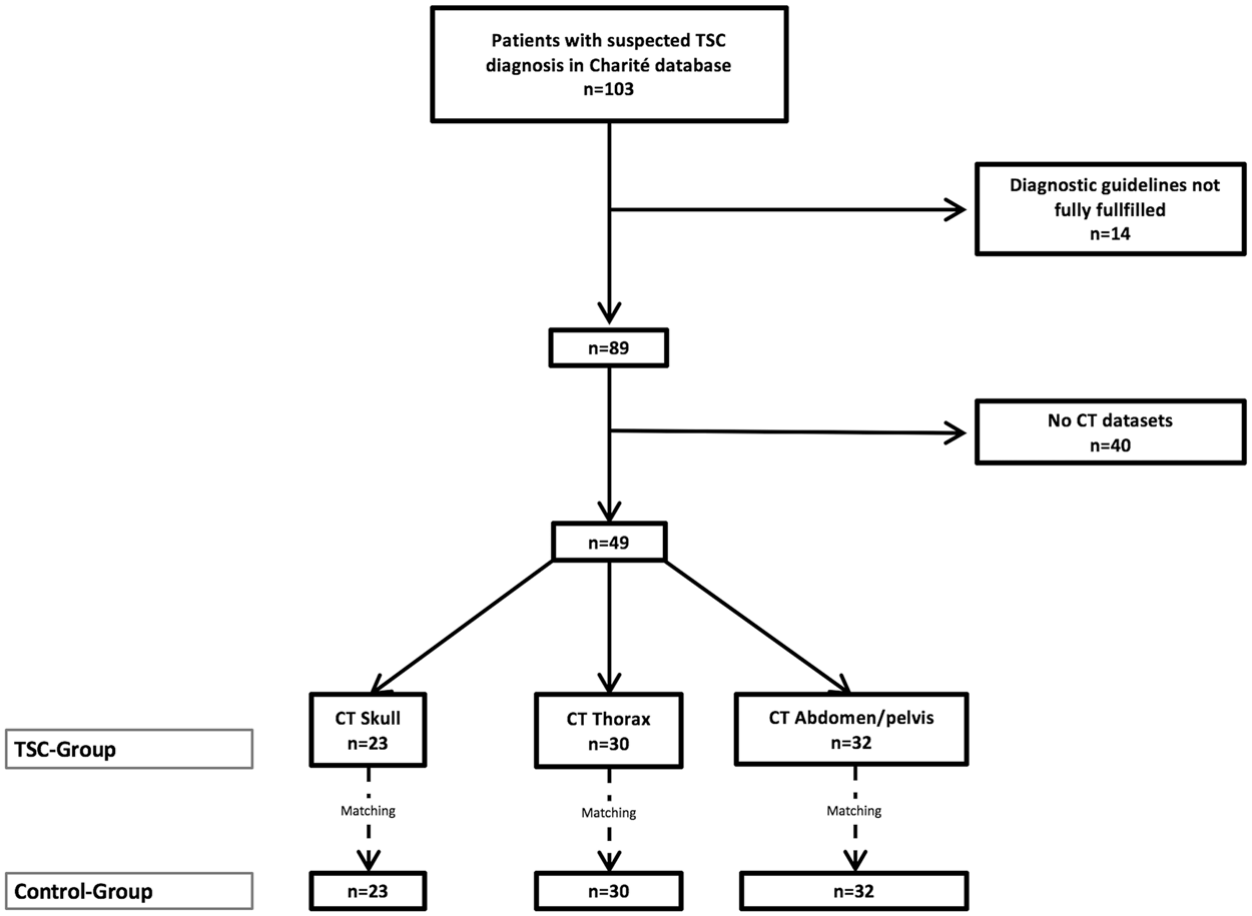
Inclusion and exclusion criteria. Overall, 89 patients with the definite TSC diagnosis were recorded. For 49 patients CT data sets were available, 23 with a CT of the skull, 30 with a CT of the thorax and 32 with a CT of the abdomen/pelvis. To determine the diagnostic cutoff value, a paired sex and age matched control collective was extracted from the imaging database.

In this study, only patients with the confirmed diagnosis of TSC, based on the current consensus criteria^2^ were included. Additionally, a CT data set of the skull, thorax and/or abdomen/pelvis had to be available. For details on exclusion criteria please see Fig. 1. Based on these criteria, a study population of 49 TSC patients (31 female, age 38.6 ± 11.6 years) could be identified. Of these, 15 (30.6%) had TSC-associated lymphangioleiomyomatosis (LAM). In this patient collective, a CT of the skull was available in 23 patients (14 female patients, age 37.1 ± 12.1 years), a CT of the thorax in 30 patients (24 female patients, age 39.7 ± 12.4 years) and a CT of the abdomen/pelvis in 32 patients (22 female patients, age 40.1 ± 12.3 years). Computed tomography imaging data sets were acquired between January 1992 and January 2017.

To determine the diagnostic cutoff value, a paired sex and age matched control collective was extracted from the imaging database of the Charité. Subjects who were referred to the emergency room and in whom a trauma CT was performed were included. In all control subjects, the trauma CT did not yield suspicious findings and no previous diseases were known based on the patients’ charts at the time of discharge from the hospital.

All data were anonymized before access by the researchers. The Charité ethics committee approved the retrospective study and waived the requirement for informed consent.

### Diagnosis of tuberous sclerosis complex

All patients were older than 18 years. The diagnosis of TSC was established based on a combination of clinical and imaging findings according to the current consensus criteria^2^. These criteria include dermatologic findings, e.g. cortical dysplasias, subependymal nodules, subependymal giant cell astrocytoma; of the kidney, e.g. renal angiomyolipoma, of the lung, e.g. lymphangioleiomyomatosis (LAM), or the heart, e.g. rhabdomyoma, or hamartoma in other organ systems such as liver, spleen or intestines (Supplementary Table S1)^2^.

### Imaging protocol

CT imaging was performed with different clinical CT imaging systems (>16 slice CT systems, including Aquilion Prime, Aquilion 64, Lightspeed Ultra, and MX 16) between January 1992 and January 2017. All images were analyzed on clinically used standard 5 mm reconstructed slices.

### Imaging analysis

All images were analyzed using PACS workstations (Centricity Radiology RA1000; GE Healthcare, Little Chalfont, United Kingdom). Bone window settings with a window width of 1700 and a contrast level of 350 were used. Images were analyzed independently and randomized and blinded to all clinical information. Additionally, sizes of focal calcifications were measured on CT images. For the detection of sclerotic bone lesions, consensus reading was performed by two readers (reader 1: one year of experience in the analysis of medical images, reader 2: eight years of experience in the analysis of medical images, five years of experience in MRI of TSC patients, radiologist). Disagreements were discussed before a single final judgment was given.

### Statistical analysis

All analysis was performed with SPSS (version 24). Variables are reported as mean ± standard deviation. The Shapiro-Wilk-Test was used to test for normal distribution. To determine whether covariates such as age/sex had an influence on the significance, a univariate ANOVA with covariate control was used. To test for significance of SBL frequency in the different bone regions between TSC and control group, a univariate ANOVA with repeated measurement under covariate control was applied. The Mann-Whitney U test was used to determine differences in size of SBLs. To obtain cutoff values, sensitivities, specificities, positive and negative predictive values for the detection of SBLs a Receiver Operating Characteristic (ROC) analysis was performed. A p-value < 0.05 was considered to be statistically significant.

### Data availability

All data generated or analyzed during this study are included in this published article.

## Results

**Figure 2:**
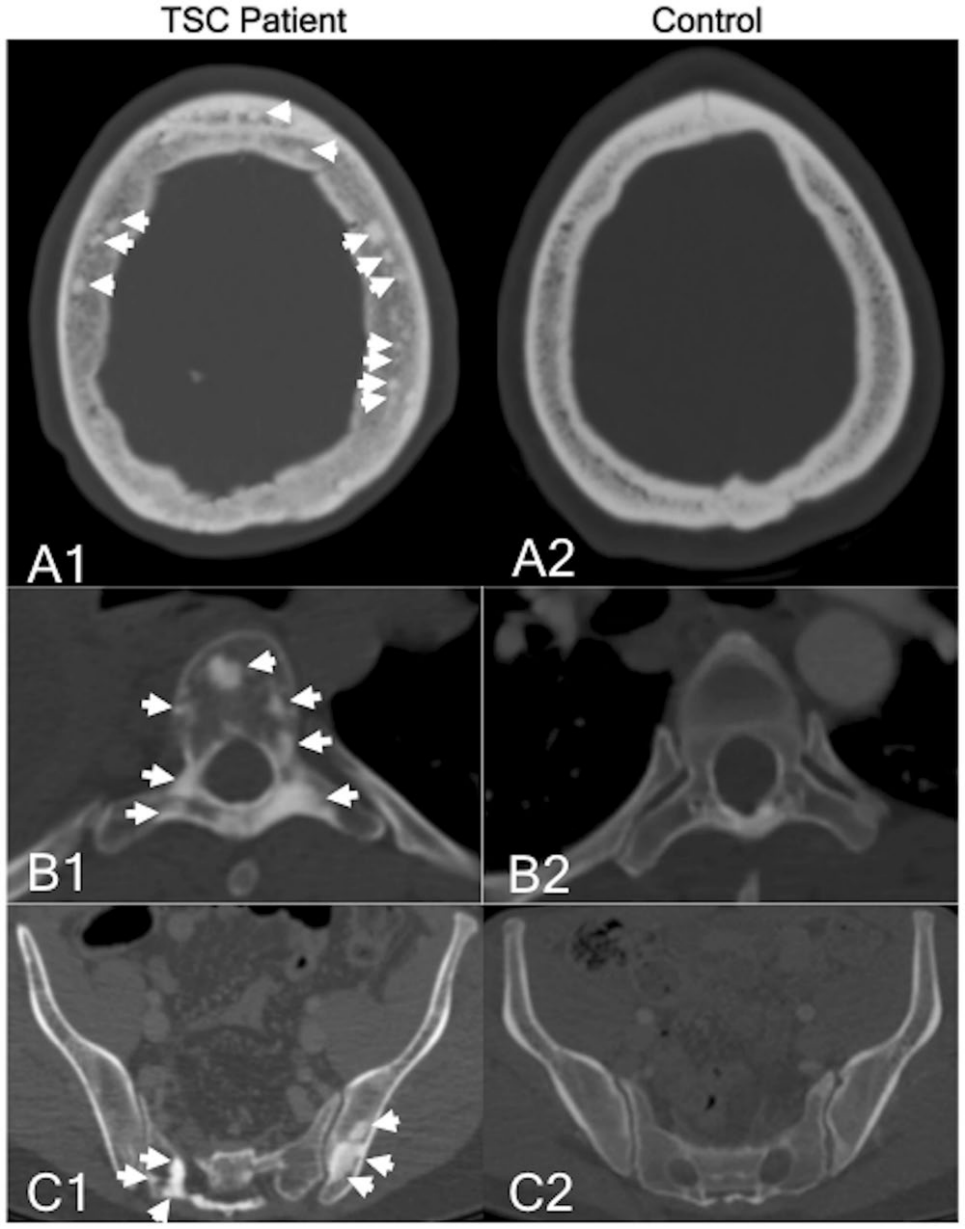
CT imaging example of the location pattern of sclerotic bone lesions in the skull, spine, and pelvis of TSC patients and control subjects. (**A1,A2)** Transversal CT of the skull of a TSC patient and a control subject. (**A1**) A relatively high number of SBLs (indicated by white arrows) can be seen in the spongiosa of the skull. The highest number of SBLs can be found in the os parietale and the os frontale. (**A2**) Normal spongiosa of a control individual without focal sclerotic bone lesions. (**B1,B2**) Transversal CT of the spine in a TSC patient and a control subject. (**B1**) The highest number of SBLs (indicated by white arrows) can be detected in the thoracic spine in the vertebral body and the arcus vertebrae/processus spinosus. Other osseous structures (e.g. sternum or ribs) were also affected, but to a lesser degree. (**B2**) Normal spongiosa of a control individual without focal sclerotic bone lesions. (**C1,C2**) Transversal CT of the pelvis in a TSC patient and a control subject. (**C1**) In the abdomen/pelvis, the second-highest number of SBLs (indicated by white arrows) could be detected in the pelvis. The highest number could be detected in the spine (example please see Fig. 2, B1). Other osseous structures such as the femur were also affected, but to a lesser degree. (**C2**) Normal spongiosa of a control individual without focal sclerotic bone lesions.

The distribution of sclerotic bone lesions (SBLs) was assessed in the complete skeletal system available by CT imaging (Table 1, Fig. 2).

**Table 1:** Distribution and frequency of SBLs of TSC patients compared to a paired control collective.

	Skull		Thorax		Abdomen	
	TSC	Control	TSC	Control	TSC	Control
Overall number of SBL’s	665	20	1427	50	1348	86
Mean number per patient	28.9	0.87*	47.6	1.67*	42.1	2.69*
Min/Max number per patient	0–172	0–4	1–207	0–5	2–165	0–8
Mean diameter (mm)	4.92	2.46*	5.44	2.227*	6.1	3.07*
Min/Max diameter (mm)	0.7–26.0	1.2–4.4	0.6–40.1	0.7–4.9	0.6–48.0	0.7–10.7

^*^p < 0.05 TSC patients vs matched control collective

TSC: Tuberous sclerosis complex; SBL: Sclerotic bone lesion.

### Distribution of sclerotic bone lesions in the skeletal system

**Figure 3:**
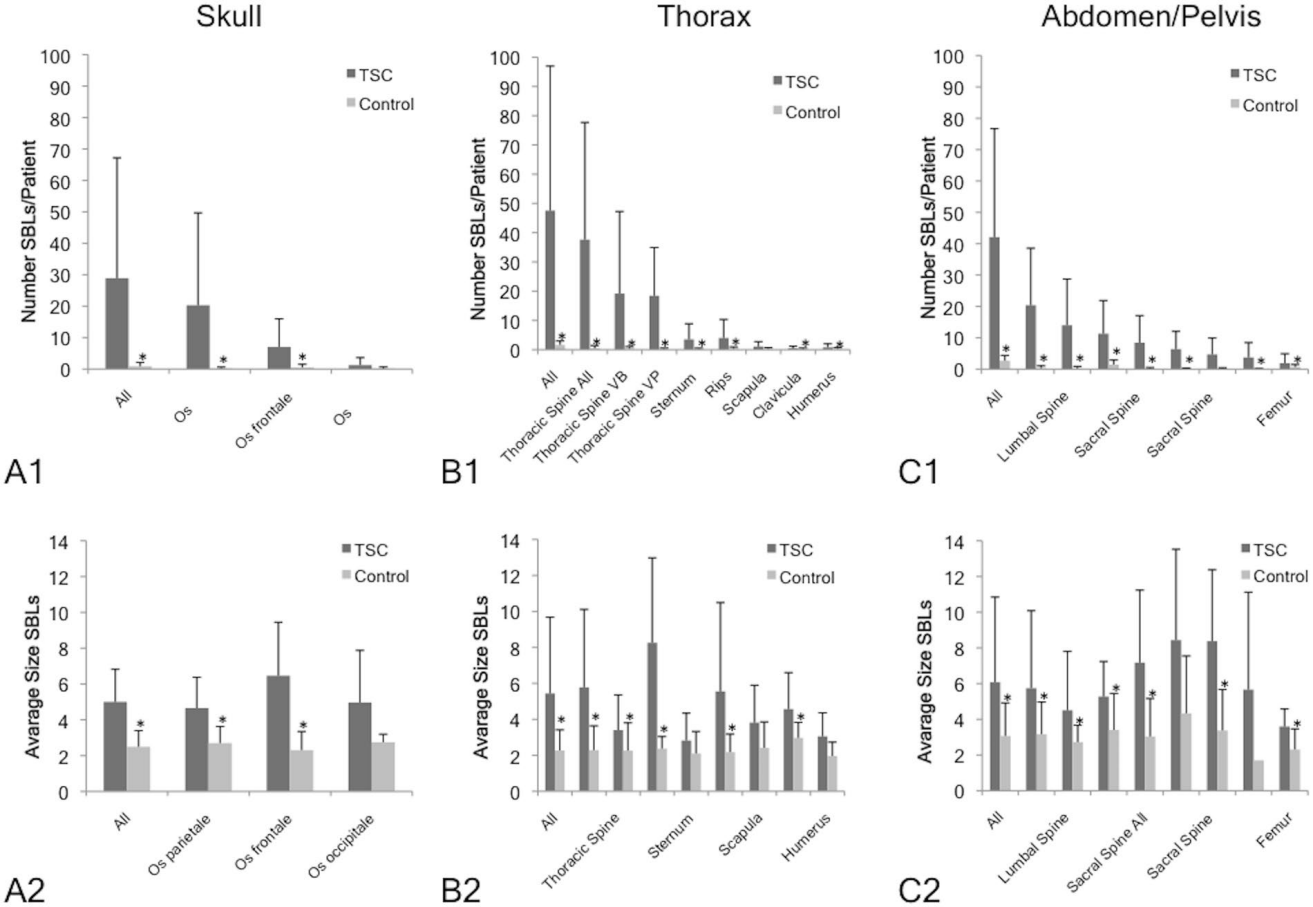
Frequency and location pattern of sclerotic bone lesions in TSC patients and control subjects. The average number of SBLs per patient and the average size of SBLs is given for the different regions investigated by CT. (**A**) In the CT scan of the skull, the highest number of SBLs per TSC patient was found in the os parietale and os frontale, while only a small number of SBLs was found in the control collective (**A1**) The size of SBLs did not differ significantly between the different regions (**A2**). (**B**) The overall highest number of SBLs per TSC patient was found in the CT of the thorax (**B1**). The frequency of SBLs was highest in the thoracic spine. Besides the thoracic spine, SBLs also occurred relatively frequently in the sternum and ribs (**B1**). The average size of SBLs of TSC patients in the spine was significantly higher compared to the control collective (**B2**). (**C**) A relatively high number of SBLs per TSC patients was found in the abdomen/pelvis (**C1**). The highest frequency of SBLs was observed in the spine, followed by the pelvis and the femur. The frequency of SBLs in the control collective was relatively low. Size measurements differed significantly between TSC patients and the control (**C2**). *Indicates a significant difference (p < 0.05) between TSC patients and the matched control collective.

A total of 3439 SBLs were detected in all analyzed regions on CT. In the matched control collective, 157 SBLs could be identified. For details regarding the distribution and size of SBLs please refer to Fig. 3.

### Frequency, size and location pattern of sclerotic bone lesions in the skull

**Figure 4:**
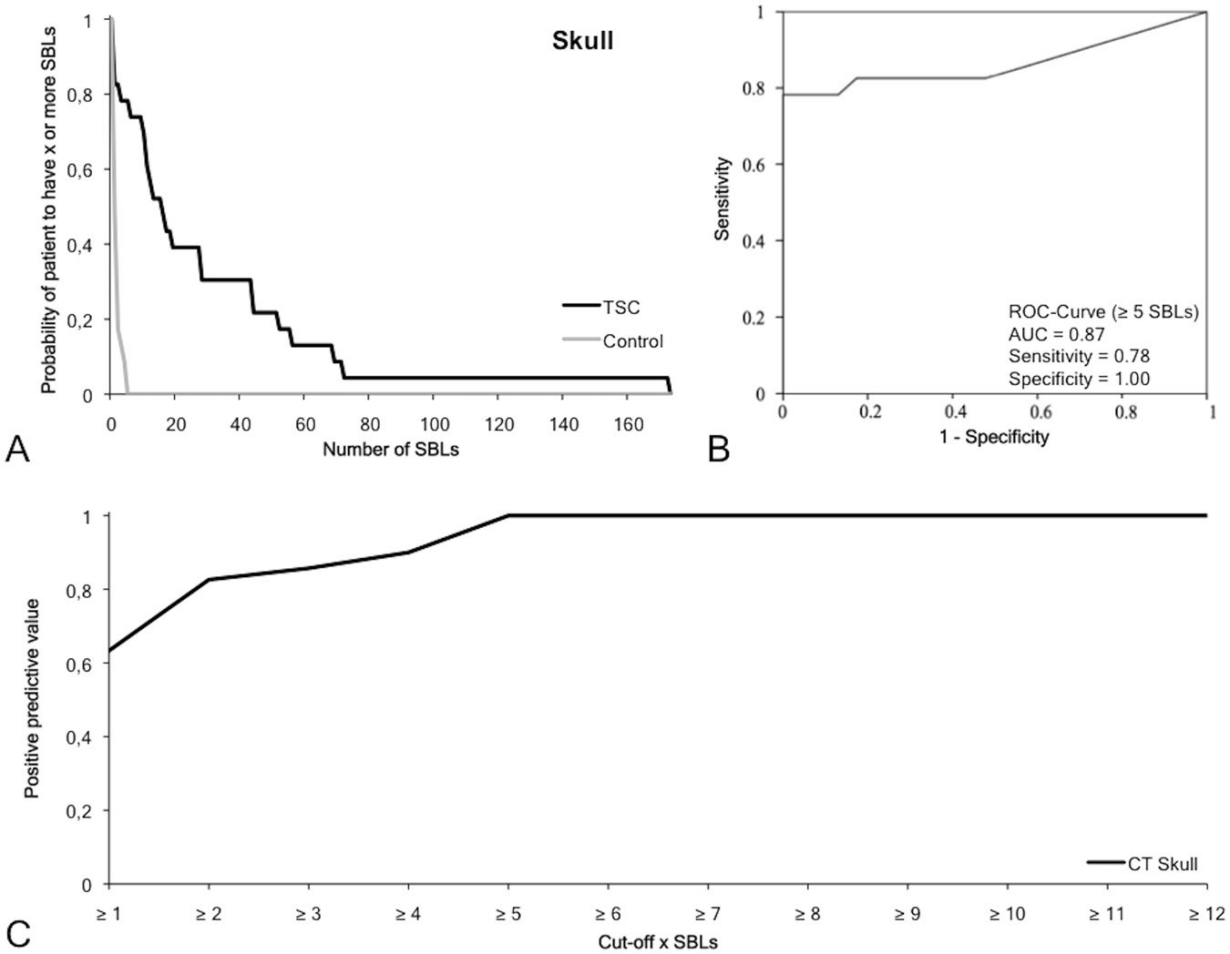
Diagnostic value of sclerotic bone lesions in the skull. (**A**) The graph demonstrates the probability of subjects to have X or more SBLs depending on the overall number of SBLs in that region. In the TSC group, the number of SBLs is relatively high and varies between the subjects. In the control group the probability drops to 0 if a threshold of five SBLs is reached. (**B**) ROC curve with a cutoff of five SBLs demonstrates a high sensitivity (0.78), specificity (1) and AUC (0.87) for the differentiation of TSC patients from controls. (**C**) Positive predictive value for a positive TSC diagnosis the specific SBL cutoff values. SBLs: Sclerotic bone lesion. ROC: Receiver operating characteristics.

In the skull, 665 SBLs (Figs 2 and 4) could be detected in TSC patients (n = 23), whereby a specific location pattern was found. Most lesions were detected in the os parietale with 20.3 ± 29.4 SBLs per patient. The second highest number of SBLs was detected in the os frontale with 7.0 ± 8.9 SBLs per patient. In the other bone regions, including the os occipitale and the os temporale, only a relatively low number of SBLs could be detected. The average size of SBLs was 5.0 ± 1.82 mm. A significantly (p ≤ 0.05) larger size of SBLs was measured in the os frontale compared to the os parietale. In the matched control collective, 20 SBLs were identified without specific location pattern. The average SBL number in the control collective was 0.9 ± 1.3 SBLs per patient with a size of 2.7 ± 0.85 mm. For the os parietale and os frontale, the frequency and size of SBLs was significantly (p < 0.05) higher for TSC patients compared to the control group.

### Frequency, size and location pattern of sclerotic bone lesions in the thorax

**Figure 5:**
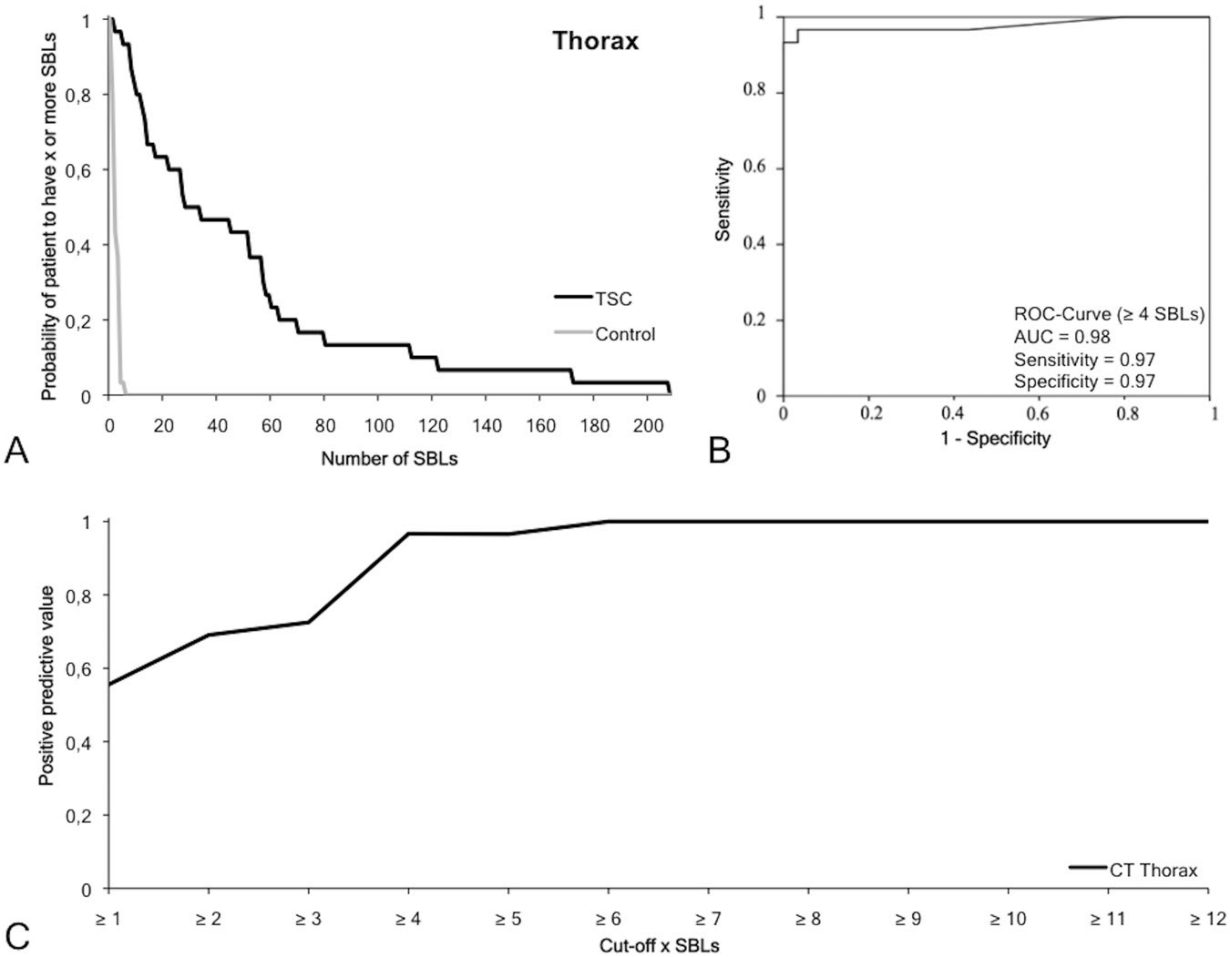
Diagnostic value of sclerotic bone lesions in the thorax. (**A**) The graph demonstrates the probability of subjects to have X or more SBLs depending on the overall number of SBLs in that region. In the TSC group the number of SBLs is relatively high and varies between the subjects. In the control group the probability drops to 0 if a threshold of five SBLs is reached. (**B**) ROC curve with a cutoff of five SBLs demonstrates a high sensitivity (0.94), specificity (0.91) and AUC (0.97) for the differentiation of TSC patients from controls. (**C**) Positive predictive value for a positive TSC diagnosis the specific SBL cutoff values. SBLs: Sclerotic bone lesion. ROC: Receiver operating characteristics.

In the thorax, 1426 SBLs (Figs 2 and 5) could be identified in TSC patients (n = 30), whereby most lesions were detected in the thoracic spine with 37.6 ± 40.0 SBLs per patient (19.2 ± 28.0 vertebral body and 18.4 ± 16.5 arcus vertebrae/processus spinosus). The size of SBLs in the arcus vertebrae/processus spinosus was significantly larger (p ≤ 0.05) compared to the vertebral body (7.3 ± 2.5 vs. 3.8 ± 3.0 mm). The second highest number of SBLs was detected in the ribs with 4.0 ± 6.3 SBLs per patient. The third highest number of SBLs was detected in the os sternale with 3.5 ± 5.3 SBLs per patient. In the other bone regions, including the scapula and clavicula, only a relatively low number of SBLs could be identified. The average size of SBLs was 5.3 ± 1.9 mm. SBLs of the thoracal spine were significantly (p < 0.05) larger compared to SBLs of the sternum and scapula. In controls, 50 SBLs were detected without location pattern for the different bones of the thorax. The average number in the control collective was 1.7 ± 1.3 SBLs per patient with a size of 2.3 ± 0.9 mm.

### Frequency, size and location pattern of sclerotic bone lesions in the abdomen/pelvis

**Figure 6:**
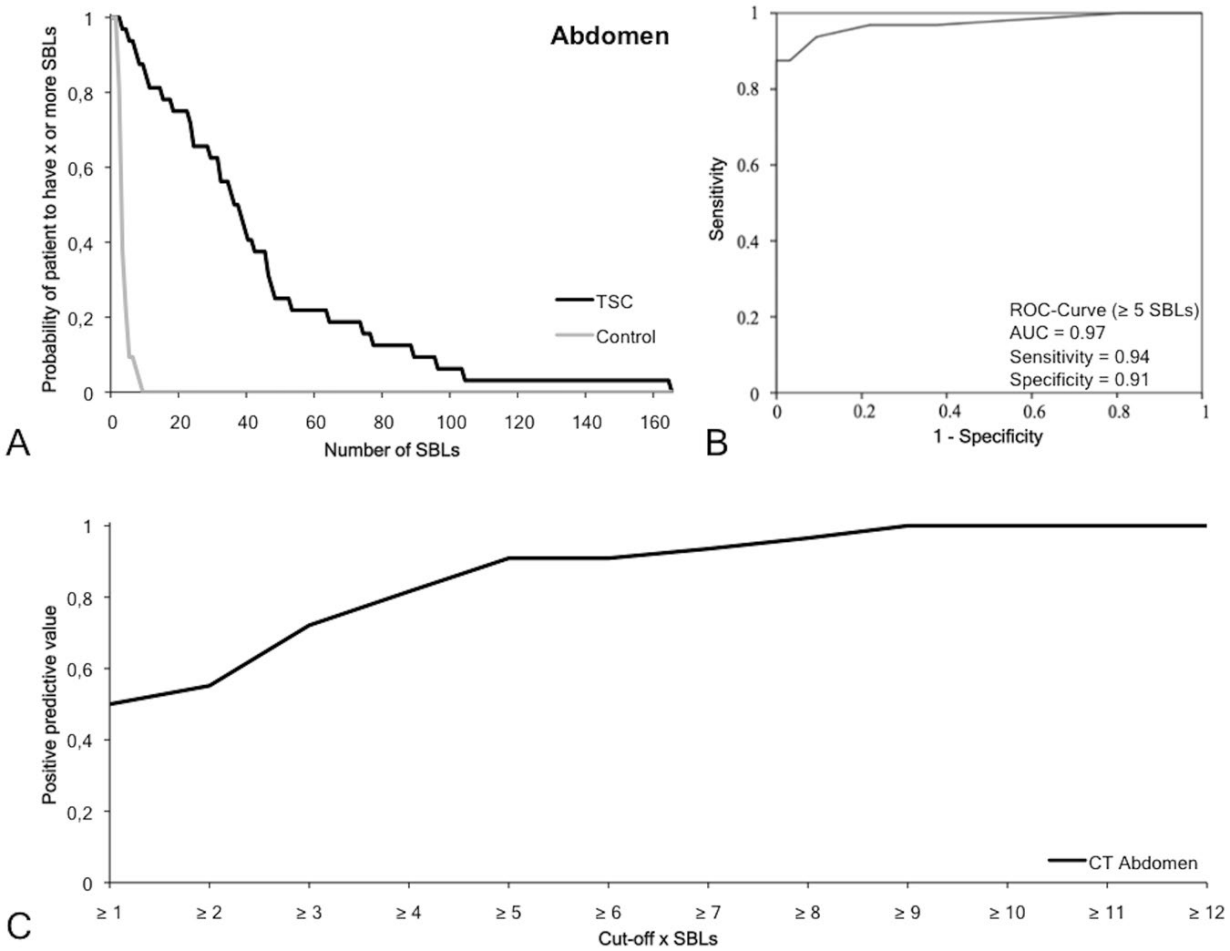
Diagnostic value of sclerotic bone lesions in the abdomen/pelvis. (**A**) The graph demonstrates the probability of subjects to have X or more SBLs depending on the overall number of SBLs in that region. In the TSC group, the number of SBLs is relatively high and varies between the subjects. In the control group, the probability drops to 0 if a threshold of five SBLs is reached. (**B**) The ROC curve with a cutoff of five SBLs demonstrates a high sensitivity (0.94), specificity (0.91) and AUC (0.97) for the differentiation of TSC patients from controls. (**C**) Positive predictive value for a positive TSC diagnosis the specific SBL cutoff values. SBLs: Sclerotic bone lesion. ROC: Receiver operating characteristics.

In the abdomen/pelvis, 1348 SBLs (Figs 2 and 6) could be detected in TSC patients (n = 32). Most of the lesions were located in the lumbal spine with 20.4 ± 18.2 SBLs per patient (14.0 ± 14.7 vertebral body and 6.4 ± 5.6 arcus vertebrae/processus spinosus). In the spine, most SBLs were detected in the lumbar part, only a smaller number was detected in the os sacrum. The second highest number of SBLs was detected in the pelvis with 11.3 ± 10.6 SBLs per patient, especially in the lateral parts of the os ilium and close to the acetabulum. SBLs were significantly larger in the os ilium compared to the os pubis und os ischii. In the matched control collective, 87 SBLs were detected with no specific distribution for the different bones of the abdomen/pelvis. The average number and size in the control collective was 2.7 ± 1.7 SBLs per patient with a size of 3.1 ± 1. 3 mm.

In all three CT regions, no significant influence (p > 0.05) of age, sex, genetic status or LAM on the number or size of SBLs could be found.

### Diagnostic potential of sclerotic bone lesions

Significant differences (p < 0.05) between TSC patients and the control collective for the overall frequency of SBLs in the different regions of the skeletal system were found (Table 1, Table 2). In the control group, a maximum of eight SBLs were detected per patient and region; in the TSC group up to 207 SBLs were found per region. Based on the number of SBLs, different diagnostic cutoffs could be calculated for the different regions.

**Table 2:** Summary of cutoff values for SBLs frequencies in the different investigated regions.

Body region	Cut-off for SBL frequency	Sensitivity	Specificity	PPV	NPV	Youden-Index
Skull	≥5	78.3%	100%	100%	82.1%	0.78
Thorax	≥4	96.7%	96.7%	96.7%	96.7%	0.93
Abdomen/Pelvis	≥5	93.8%	90.6%	90.9%	9.6%	0.84
Skull + Thorax	≥5 + ≥ 4	99.3%	100%	100%	99.4%	0.99
Skull + Abdomen/Pelvis	≥5 + ≥ 5	98.6%	100%	100%	98.9%	0.99
Thorax + Abdomen/Pelvis	≥4 + ≥ 5	99.8%	99.7%	99.7%	99.8%	0.99

PPV: Positive predictive value, NPV: Negative predictive value, SBL: Sclerotic bone lesion.

In the skull, a frequency of five or more SBLs yielded the optimal cutoff value for a reliable diagnosis of TSC (Table 2, Fig. 4). Based on this SBL frequency, a sensitivity of 0.78, a specificity of 1, a positive predictive value of 1 and a negative predictive value of 0.82 with a Youden-Index of 0.78 could be calculated. The area under the curve was 0.87.

In the thorax, a frequency of four or more SBLs yielded the optimal cutoff value (Table 2, Fig. 5) with a sensitivity of 0.97, a specificity of 0.97, a positive predictive value of 0.97 and a negative predictive value of 0.97 with a Youden-Index of 0.93 and an area under the curve of 0.98.

In the abdominal/pelvis, a frequency of five or more SBLs yielded the optimal cutoff value (Table 2, Fig. 6) with a sensitivity of 0.94, a specificity of 0.91, a positive predictive value of 0.91 and a negative predictive value of 0.94 with a Youden-Index of 0.84. The area under the curve was 0.97.

The combination of SBL frequencies from two imaging regions resulted in a further improvement of sensitivities and specificities (Table 2). If SBL frequency data from skull and thorax were combined, SBL cutoff values of ≥5 and ≥4 resulted in a sensitivity of 0.99 and a specificity of 1 (AUC 0.99). If SBL frequency data from skull and abdomen/pelvis were combined, with SBL cutoff values of ≥5 and ≥5, a sensitivity of 0.99 and a specificity of 1 was achieved (AUC 0.99). If SBL frequency data from thorax and abdomen/pelvis were combined, with SBL cutoff values of ≥4 and ≥5, a sensitivity of 1 and a specificity of 1 was achieved (AUC 0.99). It is important to mention that no influence of age or sex on the frequency or distribution of SBLs was found.

## Discussion

This study demonstrated that diagnosis of TSC may be suspected based on the frequency, size and location pattern of sclerotic bone lesions (SBLs). If more than four SBLs were detected in a CT scan of any main body region, the differential-diagnosis of TSC has to be considered.

In the skull, a sensitivity of 0.78 and specificity of 1, in the thorax a sensitivity of 0.97 and a specificity of 0.97, and in the abdomen/pelvis a sensitivity of 0.94 and a specificity of 0.91 was achieved based on region specific cutoff values for the SBL frequency compared to controls. If composite scores were used, even higher sensitivities and specificities were achieved. As frequency and location pattern of SBLs were independent of age and sex of TSC patients, they could represent a potential imaging biomarker for the diagnosis of TSC especially as an index finding in CT scans.

### Affection of the osseous system in patients with TSC

Different previous reports have already suggested that the osseous system of TSC patients is affected. The association of TSC with bone cysts and focal sclerotic lesions has been described in case reports and smaller studies, which were published as early as in the 1960s^6–8^. One of the earliest studies in this context reported that bone cysts develop predominantly in smaller periphery bones of the hands or feet^20^. In more central bones they are usually not detectable^18^. Bone cysts have initially been introduced to the diagnostic TSC guidelines as a minor criterion. However, this feature was found to be relatively unspecific and was therefore removed from the revised diagnostic TSC-guidelines in 2012^2,21^.

In contrast, increasing evidence is accumulating that sclerotic bone lesions could be relevant for the diagnosis of TSC^18^. The exact etiology of SBLs is currently not fully elucidated. It is however thought that SBLs represent a form of hamartoma, comparable to other TSC associated brain or skin hamartomas. Currently, no clear evidence regarding the development or histopathology of SBLs is available^6^.

From a diagnostic perspective, sclerotic bone lesions (SBLs) in TSC patients are easy to detect on CT scans and morphologically comparable to focal bone islands or so-called enostoses. These lesions represent compact dense bone tissue localized in the medullary cavity of bones^7^. As focal bone islands have no known pathological role, their prevalence or anatomic distribution has not been clearly defined in the general population. It is however important to mention that focal sclerotic lesions in bones can also be associated with different diseases, including osteoblastic metastasis, mastocytosis and osteopoikilosis^22^. For these entities a detailed analysis of location, size and total frequencies of SBLs has not been performed, so far^23^.

Previous case reports and studies have already reported the association of sclerotic bone lesions with TSC^6–8^. A recent study by Avila *et al*. investigated a cohort of patients with TSC-associated lymphangioleiomyomatosis (LAM)^18^. In this study, the detailed analysis of the frequency/distribution of SBLs was limited to a relatively small subgroup of 15 mostly female patients without control collective.

In contrast, the present study defines cutoff values allowing a differentiation of TSC patients from control individuals. With regard to the location pattern of SBLs, the most promising locations to specifically search CT datasets for SBLs are the os parietale, frontale and occipitale, the spine, sternum, ribs and pelvis.

### Pathways for the diagnosis of tuberous sclerosis complex (TSC)

TSC patients with a sporadic mutation account for up to 80% of the overall TSC cohort. In these patients, the TSC diagnosis is usually established on the basis of clinical signs that are diagnosed incidentally. These include typical skin lesions or clinical symptoms that require medical attention, e.g. seizures. As a result of the highly variable clinical picture and a relatively large proportion of oligosymptomatic patients, it is assumed that a high number of TSC patients remains undiagnosed until adulthood when complications – like acute AML bleeding - point to the underlying disease^4,17^.

In a clinical setting, TSC is currently diagnosed based on specific diagnostic guidelines^2^. If TSC is suspected, systematic clinical and imaging investigations are performed with regard to these criteria to establish the diagnosis. Genetic analysis may also be helpful, especially in inherited cases or for genetic counselling, but is not required for diagnosing TSC and positive in only up to 80% of patients^2^. Most of the clinical signs of TSC – and therefore the diagnostic criteria – are age dependent, which can make the diagnosis more challenging. Potential cerebral and heart manifestations develop during early infancy, whereas renal and skin manifestations may occur later during childhood^3,24^. Pulmonary manifestations (LAM) can be detected from late adolescence on^16^.

SBLs as a potential imaging biomarker could be especially important with regard to the diagnostic workup of the following patient collectives: First, for oligosymptomatic patients and/or younger patients, who do not fully meet the diagnostic TSC guideline criteria. Second, as an index finding in all patients with undiagnosed TSC, in which a CT scan is performed due to other clinical indications. In these patients, the recognition of SBLs as a typical imaging sign of TSC could represent the crucial connection towards a timely diagnosis and, if indicated, a specific therapy. Therefore, pediatric radiologists should also be aware of SBLs in TSC patients, however, frequency of SBLs in children has yet to be investigated.

The TSC population investigated in this study is unique and has several advantages compared to previously performed studies^18^. In the current study, a relatively large adult TSC patient cohort - especially given the low prevalence of TSC - with a balanced distribution of sex and age was investigated. All patients were followed up long-term and had a confirmed diagnosis of TSC. CT scans from all relevant body regions were available for analysis.

### Limitations of this study

Calcifications were not confirmed histologically. Due to radiation exposure, whole-body CT-investigations of the total TSC cohort were not available in all patients. Only adult patients were included in our study. The development of SBLs during childhood was not investigated due to the radiation exposure associated with CT. Results were not compared to other disease entities potentially associated with SBLs such as mastocytosis and osteopoikilosis.

## Conclusion

This study demonstrates that a potential diagnosis of TSC can be suspected based on the frequency, size and location pattern of sclerotic bone lesions in adult patients. As a result, SBLs could serve as a potential imaging biomarker in the diagnostic guidelines for TSC. The reliable diagnosis of TSC is of high clinical relevance, as novel therapies can be initiated to treat organ manifestations and prevent complications of TSC.

## Electronic supplementary material

Supplementary Table S1
