# Supplementary material for: Sclerotic bone lesions as a potential imaging biomarker for the diagnosis of tuberous sclerosis complex
Source: Sci Rep. 2018 Jan 17;8:953. doi: 10.1038/s41598-018-19399-7 (PMC5772483; doi:10.1038/s41598-018-19399-7)
Supplement: Supplementary file 1 — Supplementary Table S1 [file 41598_2018_19399_MOESM1_ESM.pdf]

Sclerotic bone lesions as a potential imaging biomarker for the diagnosis of tuberous sclerosis complex

Susanne Brakemeier[1*], Lars Vogt[2], Lisa C Adams[2], Bianca Zukunft[1],

Gerd Diederichs[2], Bernd Hamm[2], Klemens Budde[1], Kai-Uwe Eckardt[1], Marcus R Makowski[2,3]

Supplementary Table

Diagnostic criteria for TSC

A. Genetic diagnostic criteria

The identification of either a TSC1 or TSC2 pathogenic mutation in DNA from normal tissue is sufficient to make a definite diagnosis of tuberous sclerosis complex (TSC).

Note that 10% to 25% of TSC patients have no mutation identified by conventional genetic testing, and a normal result does not exclude TSC, or have any effect on the use of clinical diagnostic criteria to diagnose TSC.

B. Clinical diagnostic criteria

Major features

1. Hypomelanotic macules (≥3, at least 5-mm diameter)
2. Angiofibromas (≥3) or fibrous cephalic plaque
3. Ungual fibromas (≥2)
4. Shagreen patch
5. Multiple retinal hamartomas
6. Cortical dysplasias (Includes tubers and cerebral white matter radial migration lines)
7. Subependymal nodules
8. Subependymal giant cell astrocytoma
9. Cardiac rhabdomyoma
10. Lymphangioleiomyomatosis (LAM)* 11. Angiomyolipomas (≥2)*

*A combination of the two major clinical features (LAM and angiomyolipomas) without other features does not meet criteria for a definite diagnosis.

Minor features

1. “Confetti” skin lesions
2. Dental enamel pits (>3)
3. Intraoral fibromas (≥2)
4. Retinal achromic patch
5. Multiple renal cysts
6. Nonrenal hamartomas

Definite diagnosis: Two major features or one major feature with ≥2 minor features

Possible diagnosis: Either one major feature or ≥2 minor features

Adopted from [2]
